# A Cotraining-Based Semisupervised Approach for Remaining-Useful-Life Prediction of Bearings

**DOI:** 10.3390/s22207766

**Published:** 2022-10-13

**Authors:** Xuguo Yan, Xuhui Xia, Lei Wang, Zelin Zhang

**Affiliations:** 1 Key Laboratory of Metallurgical Equipment and Control Technology, Ministry of Education, Wuhan University of Science and Technology, Wuhan 430081, China; 2 Hubei Key Laboratory of Mechanical Transmission and Manufacturing Engineering, Wuhan University of Science and Technology, Wuhan 430081, China; 3 Precision Manufacturing Institute, Wuhan University of Science and Technology, Wuhan 430081, China

**Keywords:** RUL prediction, cotraining, semisupervised learning, bearings

## Abstract

The failure of bearings can have a significant negative impact on the safe operation of equipment. Recently, deep learning has become one of the focuses of RUL prediction due to its potent scalability and nonlinear fitting ability. The supervised learning process in deep learning requires a significant quantity of labeled data, but data labeling can be expensive and time-consuming. Cotraining is a semisupervised learning method that reduces the quantity of required labeled data through exploiting available unlabeled data in supervised learning to boost accuracy. This paper innovatively proposes a cotraining-based approach for RUL prediction. A CNN and an LSTM were cotrained on large amounts of unlabeled data to obtain a health indicator (HI), then the monitoring data were entered into the HI and the RUL prediction was realized. The effectiveness of the proposed approach was compared and analyzed against individual CNN and LSTM and the stacking networks SAE+LSTM and CNN+LSTM in the existing literature using RMSE and MAPE values on a PHM 2012 dataset. The results demonstrate that the RMSE and MAPE value of the proposed approach are superior to individual CNN and LSTM, and the RMSE value of the proposed approach is 54.72, which is significantly lower than SAE+LSTM (137.12), and close to CNN+LSTM (49.36). The proposed approach has also been tested successfully on a real-world task and thus has strong application value.

## 1. Introduction

The prognostics and health management (PHM) technique has recently been studied in terms of assessing and managing the health status of equipment or components with the help of statistical algorithms or models using large quantities of condition-monitoring data and information [1]. The PHM technique can predict potential failures in advance and combine various equipment or component information to make maintenance decisions, thereby improving the safety of production processes and reducing maintenance costs [2]. RUL prediction is the most challenging technique in PHM [3]. It can be defined as the time interval during which equipment or components can be properly used continuously [4]. In this paper, we study bearings, whose failure can have a significant negative impact on the safe operation of equipment. Therefore, it is urgent and necessary to develop an effective RUL prediction approach to estimate their RUL under multiple operational conditions.

Existing RUL prediction techniques mainly include model-based and data-driven approaches [5]. Model-based approaches describe the degradation stages of equipment or components by determining an accurate physical or mathematical model [6]. However, a number of complex equipment or components render it arduous to define an accurate model due to multiple operating environments. With the rapid development of artificial intelligence and machine learning technologies, data-driven approaches have become a remarkable tool for RUL prediction. These approaches are based on data fusion and feature extraction of monitoring data and history sensor data from various degradation stages of equipment or components to establishing the mapping relationship between monitoring data and RUL [7]. The approaches do not require a priori knowledge, but are based on existing data and use individual analytical processing to mine implicit associations in the data to perform predictive operations [8]. The model construction of a health indicator (HI) that matches the degradation trend of equipment or components is central to data-driven RUL prediction approaches.

Model construction methods for RUL prediction mainly include statistical modeling and traditional machine-learning and deep-learning methods [9]. Recently, deep learning has become one of the focuses of RUL prediction due to its potent scalability and nonlinear fitting ability [10,11,12]. Early studies in this area relied on large quantities of training data for model learning. However, in real-world cases, the operation conditions of most complex equipment or components may change and the data distribution shows some variability. This will lead to a sharp drop in the performance of RUL prediction due to the poor robustness and generalization of the model [13]. The usual solution to this problem is to retrain or fine-tune the model parameters, which is a supervised learning process that requires a significant quantity of labeled data for training to improve learning performance. Unfortunately, in real-world cases, there are usually abundant unlabeled data available, but few labeled data [14]. The data labeling process of supervised learning, moreover, can be expensive and time-consuming.

Semisupervised learning can reduce the quantity of required labeled data through exploiting the available unlabeled data in supervised learning to boost the accuracy. It can address the poor generalization problem of supervised learning when there are abundant unlabeled data available, but only a few labeled samples [15]. Cotraining is one of the major semisupervised learning paradigms that iteratively trains two classifiers on two different views, and uses the predictions of either classifier on the unlabeled examples to augment the training set of the other [16]. Cotraining can obtain higher accuracy than traditional supervised learning methods due to the ability to combine multiple views of similar samples and prediction results on multiple classifiers [17]. Nowadays, cotraining and its improved approaches have been successfully applied to natural language processing [18], pattern recognition [19] and other fields. However, comparatively few publications are available about cotraining-based approaches for RUL prediction.

In this study, we innovatively adopt cotraining to RUL prediction of mechanical components. In order to better evaluate the improvement within the existing approaches, CNN and LSTM are selected as the initial network model. Individual CNN and LSTM, and the stacking networks SAE+LSTM and CNN+LSTM are selected for comparison with a PHM 2012 dataset. Since various indicators can evaluate the performance of RUL prediction, in this paper, RMSE and MAPE values are adopted to evaluate the prediction results and RMSE values are selected for comparison. We suppose that through cotraining, more accurate RUL prediction results can be achieved by fully using the deep feature extraction capability of CNN and the long-term memory capability of LSTM for time series data. The main contributions of this paper are as follows:

- We propose a semisupervised learning approach for RUL prediction to address the problem that there are usually abundant unlabeled data available, but few labeled data in the actual production scenario.
- We found little literature available on cotraining for RUL prediction. We innovatively propose a cotraining-based approach for RUL prediction of bearings. A CNN and an LSTM were cotrained on large quantities of unlabeled data to obtain the HI of the bearings, then the monitoring data can be input into the HI to realize the RUL prediction.
- We conducted experiments on open datasets (PHM 2012) and real datasets of gearbox bearings in Wuhan Iron and Steel Company, China. The experimental results verify the effectiveness of the proposed approach.

The remainder of this study is organized as follows. Related work is discussed in Section 2. The proposed cotraining-based RUL prediction approach is presented along with a detailed description of each step in Section 3. The experimental setup and results are presented in Section 4. Finally, Section 5 presents the conclusions and suggests some possible avenues for further research.

## 2. Related Work

**Figure 1:**
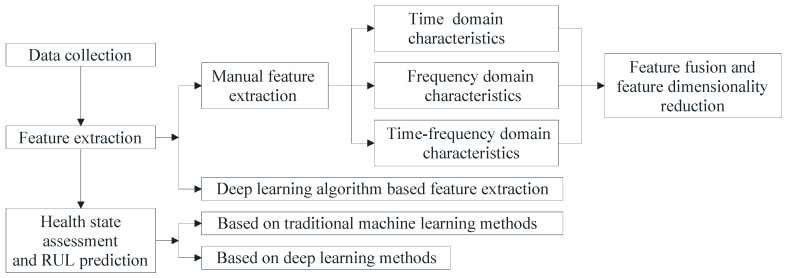
Processes and associated algorithms of data-driven RUL prediction approach.

In this section, we focus on the current state of research on feature extraction and RUL prediction model construction in the data-driven RUL prediction approach. An overview of data-driven RUL prediction process and associated algorithms is given in Figure 1.

One of the key steps in RUL prediction is to extract effective degradation features from original signals. The accuracy of RUL prediction is determined by the quality of feature extraction. Vibration signal extraction is the most widely used feature extraction technique to obtain the health status of equipment or components. It extracts the feature indicators from the vibration signal’s time domain, frequency domain, and time–frequency domain. Time-domain signal processing algorithms mainly include correlation analysis [20] and time-domain statistical analysis [21]. Frequency-domain signal processing algorithms mainly include spectrum analysis [22], cepstrum analysis [23], envelope analysis [24], order ratio spectrum analysis [25], and holographic spectrum analysis [26]. Time–frequency domain signal processing algorithms mainly include short-time Fourier transform [27], Wigner–Ville distribution [28], empirical mode decomposition methods [29] and wavelet transform [30]. These algorithms are based on manual feature extraction, which frequently require some prior knowledge and experience. The features extracted by these algorithms are mostly low-level features. In recent years, deep learning has shown its unique potential and advantages in feature extraction. Many scholars have applied deep learning to the field of signal feature extraction. Hinchi and Tkiouat [31], for example, employed a CNN model to extract rolling bearing vibration signal features and achieved improved results in feature extraction. However, these CNN-based feature extraction algorithms require a large quantity of labeled data for model monitoring and adjustment, but the data labeling process in real-world scenarios can be expensive and time-consuming. In order to solve this problem, Liu [32] provided an unsupervised deep neural network through exploiting unlabeled data to extract high-level vibration signal features of rolling bearings and achieved certain results.

RUL prediction model construction in the data-driven RUL prediction approach mainly includes traditional machine-learning and deep-learning methods. Traditional machine learning-based RUL prediction methods estimate equipment or components’ RUL by identifying patterns of variation from a large quantity of monitoring data. For example, Fumeos [33] developed an online correlation vector regression model and optimized the model for RUL prediction of bearings using heuristic algorithms. The prediction model based on traditional machine learning can meet the needs of RUL prediction; however, it does not consider the deep-level mapping relationship between degradation features and health status, resulting in a lack of generalization ability of the model. The RUL prediction model constructed by deep-learning methods can solve this problem. Many scholars have applied deep learning to the field of model construction for RUL prediction. Representative network models include: BP neural networks [34], extreme learning machine (ELM) [35], CNN [36], LSTM [37] and deep stacking network model of CNN and LSTM. In recent years, deep stacking network models of CNN and LSTM have received increasing attention from scholars due to their ability to handle chronological and spatial relationships of degradation signals. For example, Mao [38] used the Hilbert–Huang transform to extract time–frequency domain information in vibration signals and used this information as a label for whether the data were in a fault state and trained the information by CNN. After training, the monitoring data were input into the trained CNN, and then LSTM was trained with the output of the penultimate neural layer of the CNN as the input data and the RUL as the label.

In recent years, cotraining and its improved approaches have been successfully applied to various fields. However, comparatively few publications are available about cotraining-based approaches for RUL prediction. The cotraining-based approach for RUL prediction of mechanical components is a valuable area of research.

## 3. Methods

The detail of proposed cotraining-based RUL prediction approach is presented in this section. The abundant unlabeled data can be fully used in the training process to improve the accuracy of RUL prediction.

### 3.1. Brief Introduction of Cotraining, CNN and LSTM

**Figure 2:**
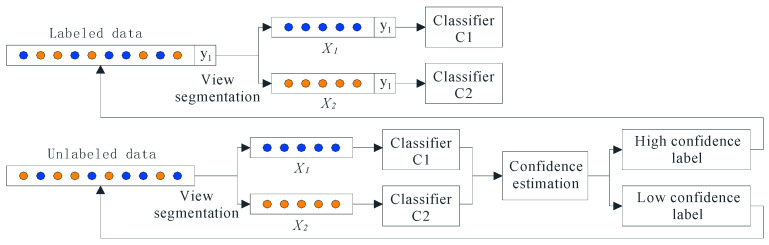
Algorithm flowchart of cotraining.

Cotraining is an effective semisupervised learning method. It uses unlabeled samples to improve prediction accuracy. In the cotraining process, random sampling is used to gradually select unlabeled samples to train classifiers [39]. An algorithm flowchart of cotraining is shown in Figure 2.

First, the labeled data are divided into two views to obtain the data representation under two different views, and two different classifiers are trained using different views as the initial classifier. Then, the initial classifier is used to estimate the label confidence of unlabeled samples, and high-confidence samples are added to the labeled data to further iterative training and optimize the classifier. When all unlabeled samples are self-labeled by the classifier, the training model ends.

Define a sample space $x=x_{1}\times x_{2}$, where $x_{1}$ and $x_{2}$ correspond to two different “views” of the same sample. The process of standard cotraining algorithm is shown in Algorithm 1:
**Algorithm1:** The standard cotraining algorithm**Input:** a set *L* of labeled training samples    a set *U* of unlabeled samples **Process:**
    Create a pool $U^{\prime }$ of samples by choosing *u* samples at random from *U*
    Loop for *k* iterations:
      Use *L* to train a classifier $h_{1}$ that considers only the $x_{1}$ portion of *x*      Use *L* to train a classifier $h_{2}$ that considers only the $x_{2}$ portion of *x*      Allow $h_{1}$ to label *p* positive and *n* negative samples from $U^{\prime }$      Allow $h_{2}$ to label *p* positive and *n* negative samples from $U^{\prime }$      Add these self-labeled samples to *L*
Randomly choose 2p+2n samples from *U* to replenish $U^{\prime }$


Step 1: Define the labeled training set *L* and the unlabeled dataset *U*;

Step 2: Randomly select *u* samples from *U* to create sample buffer pool;

Step 3: Consider two views $x_{1}$ and $x_{2}$, and train the classifiers $h_{1}$ and $h_{2}$ using *L*;

Step 4: All samples in *U* are labeled with $h_{1}$, from which *p* positive and *n* negative samples are selected with high confidence. $h_{2}$ is treated in the same way;

Step 5: Add these self-labeled samples to *L*, that is, choose p+n samples from $h_{1}$ to $x_{2}$, and choose p+n samples from $\;h_{2}$ to $x_{1}$. Then randomly choose 2p+2n samples from *U* to replenish $U^{\prime }$;

Step 6: Iterate Step 3 to Step 5 *k* times.

Cotraining starts with training both classifiers on the labeled training set, and then classifier A labels a portion of the unlabeled dataset, generating pseudolabels for the unlabeled samples. Then, samples with high labeling confidence are selected and added to the training set of classifier B. Similarly, classifier B also labels a portion of the unlabeled dataset, selects those with high labeling confidence and adds to the training set of classifier A. Labeling each other until the maximum number of iterations or no samples with high confidence are added. In this way, the training set of the classifier is expanded continuously, allowing the classifier to learn more knowledge.

In the cotraining algorithm, unlabeled sample with the highest labeling confidence is the sample that is most consistent with the labeled sample of the classifier after labeling. It is to maximize:

(1) $$\Delta_{u}=\frac{1}{\left|L\right|}\underset{x_{i}\in L}{\sum }{\left(y_{i}-h(x_{i})\right)}^{2}-\frac{1}{\left|L\right|}\underset{x_{i}\in L}{\sum }{\left(y_{i}-h^{\prime }\left(x_{i}\right)\right)}^{2}$$

in the sample set *U*, where *h* denotes the model learned by the current classifier, *L* denotes the labeled training set, $x_{i}\in L$ denotes the unlabeled samples, and $h^{\prime }$ denotes the classifier obtained by adding the *h* labeled samples to the training set and retraining them. This *Δ* function is the classifier prediction error before adding the labeled samples minus the classifier prediction error trained after adding the labeled samples. If Δ>0, it means that the performance of the classifier has improved, and the labeled sample with the largest Δ value is the one with the highest confidence.

### 3.2. Cotraining in RUL Prediction

**Figure 3:**
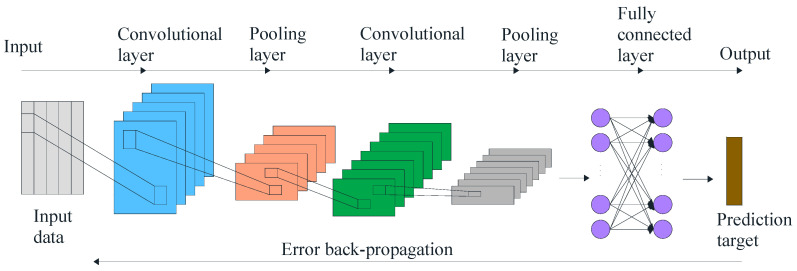
The architecture of CNN.

CNN is one of the most representative algorithms in deep learning. It has two critical structural layers: convolutional and pooling. The convolutional layer computes the convolutional operation of the input data using kernel filters to extract fundamental features. The pooling layer is usually followed to the convolutional layer. In the pooling layer, subsampling is applied to reduce the dimension and avoid overfitting. The typical architecture of CNN is shown in Figure 3.

In RUL prediction, CNN performs error back-propagation based on the BP algorithm. By combining optimization methods such as gradient descent algorithm to train the weight parameters of each layer, the local feature extraction of the input data can be achieved, and the abstract high-dimensional spatial features can be produced. It is then fitted by a fully convolutional neural network (FCN) to achieve the prediction of RUL.

**Figure 4:**
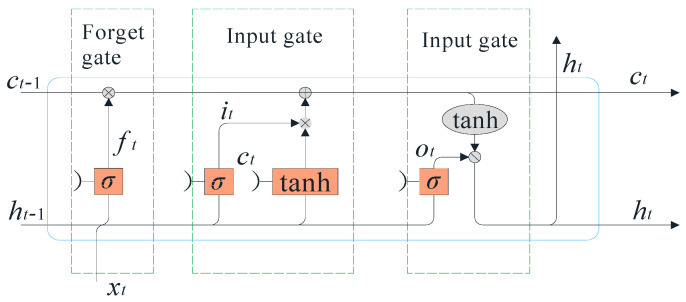
Internal structure of LSTM with forget gates.

The traditional recurrent neural network (RNN) has certain memory ability. However, when dealing with long-time series, RNN is prone to gradient explosion or disappearance and cannot learn the relevant information of input data. LSTM is a variant of RNN used in deep learning and has long-term memory ability. The architecture of an LSTM consists of units called memory cells, and the memory capacity of the cells can be improved by introducing “gates” into the cells. The LSTM cell has been transformed and generalized by many researchers in recent years, which mainly include LSTM with forget gates, LSTM without forget gates, and LSTM with peephole connections. Considering that LSTM with forget gates is the most widely used LSTM unit, this study takes it as the basic unit structure of the LSTM unit. The internal structure of this unit is shown in Figure 4. According to the figure, the internal operation process of the LSTM model is as follows:

(2) $$f_{t}=\sigma \left(W_{fh}h_{t-1}+U_{fx}x_{t}+b_{f}\right)$$

(3) $$i_{t}=\sigma \left(W_{ih}h_{t-1}+U_{ix}x_{t}+b_{i}\right)$$

(4) $$o_{t}=\sigma \left(W_{oh}h_{t-1}+U_{ox}x_{t}+b_{o}\right)$$

(5) $$c_{t}=f_{t}\cdot c_{t-1}+i_{t}\cdot {\tilde{c}}_{t}$$

(6) $$h_{t}=o_{t}\cdot \tan h\left(c_{t}\right)$$

where $f_{t}$,$\;i_{t}$, $o_{t}$ denote the forget gate, input gate and output gate at moment *t*. $c_{t}$, $h_{t}$, $x_{t}$ denote the cell state, hidden state and cell input at the moment *t*. W and U denote the weights of the hidden state and the cell input.

The forget gate decides which information from the previous cell state should be forgotten. When $f_{t}=$1, the information will be completely retained; when $f_{t}=0$, the information will be completely forgot.

**Figure 5:**
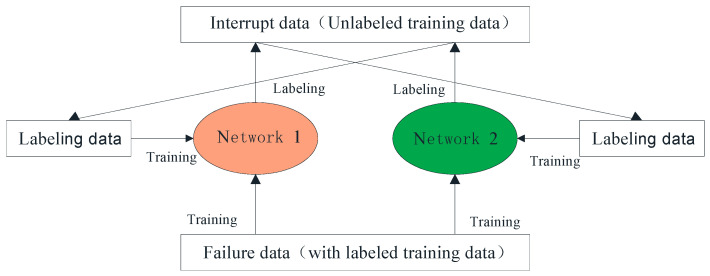
Cotraining in RUL prediction.

A general flowchart of cotraining in RUL prediction is given in Figure 5. First, two prediction networks are selected and trained separately on the failure data (labeled training data). Then, the interrupt data (unlabeled training data) are labeled, and the samples with high confidence are added to each other’s training set to expand the training set continuously. In this paper, we set CNN as the prediction Network 1 and LSTM as the prediction Network 2.

Setting CNN as the prediction Network 1 and LSTM as the prediction Network 2, the pseudocode of RUL prediction based on cotraining CNN and LSTM is as follows (Algorithm 2):
**Algorithm2:** Pseudocode of RUL prediction based on cotraining**Input**: *L*—Failure training dataset    *U*—Suspension training dataset    *T*—Maximum number of cotraining iterations    *u*—Suspension pool size
 **Training Process:** 1 *L*_1_ = *L*; *L*_2_ = *L* 2 *h*_1_ = TrainFun (*L*_1_,1); *h*_2_ = TrainFun (*L*_2_,1) 3 **Repeat** *T* times 4 Create a pool *U*′ of *u* suspension units by random sampling from *U* 5 **for** j=1 to 2 6 **for** each $X_{u}\subset U^{\prime }$ 7 $L_{u}^{p}=h_{j}\left(X_{u}\right)$ 8 $h_{j}^{\prime }=TrainFun\left(L_{j}\cup \left\{X_{u},L_{u}^{P}\right\},j\right)$; 9 $\Delta_{j,X_{u}}=\sum {\left(L_{i}^{T}-h_{j}\left(X_{i}\right)\right)}^{2}-\sum {\left(L_{i}^{T}-h_{j}^{\prime }\left(X_{i}\right)\right)}^{2}$ 10 **end** 11 **if** there exists an $\Delta_{j,X_{u}}>0$ 12 $X_{j}^{*}={\arg\;\max}_{X_{u}\subset U^{\prime }}\;\left(\Delta_{j,X_{u}}\right)$;$L_{j}^{*}=h_{j}\left(X_{j}^{*}\right)$; 13 $\pi_{j}=\left\{\left(X_{j}^{*},\;L_{j}^{*}\right)\right\}$; $U^{\prime }=U^{\prime }\backslash \pi_{j}^{*}$; 14 **else** 15 $\pi_{j}=\emptyset$; 16 **end** 17 **end** 18 **if** $\pi_{1}==\emptyset \;\&\&\;\pi_{2}==\emptyset \;exit$ 19 **else** $L_{1}=L_{1}\cup \pi_{2}$; $L_{2}=L_{2}\cup \pi_{1}$; 20 $h_{1}=TrainFun\left(L_{1},\;1\right)$; $h_{2\;}=TrainFun\left(L_{2},\;2\right)$; 21 **end**
 **Testing Process** 22 $L^{P}=\omega_{1}h_{1}\left(X\right)+\omega_{2}h_{2}\left(X\right)$ for any test data **X**

After obtaining the final prediction result $L^{P}=\omega_{1}h_{1}\left(x\right)+\omega_{2}h_{2}\left(x\right)$, the sequential quadratic programming (SQP) method can be used to optimize the ensemble weights of different prediction networks. The formula is as follows:

(7) $$\min\;E=\sum_{x_{i\in \psi }}{\left(L_{i}-\left(\omega_{1}h_{1}\left(x_{i}\right)\right)+\omega_{2}h_{2}\left(x_{i}\right)\right)}^{2}\;s.t.\;\{\begin{array}{c} \omega_{1}+\omega_{2}=1\\ 0\leq \omega_{1}\leq 1\\ 0\leq \omega_{2}\leq 1 \end{array}$$

### 3.3. The Bearing RUL Prediction Process Based on Cotraining

**Figure 6:**
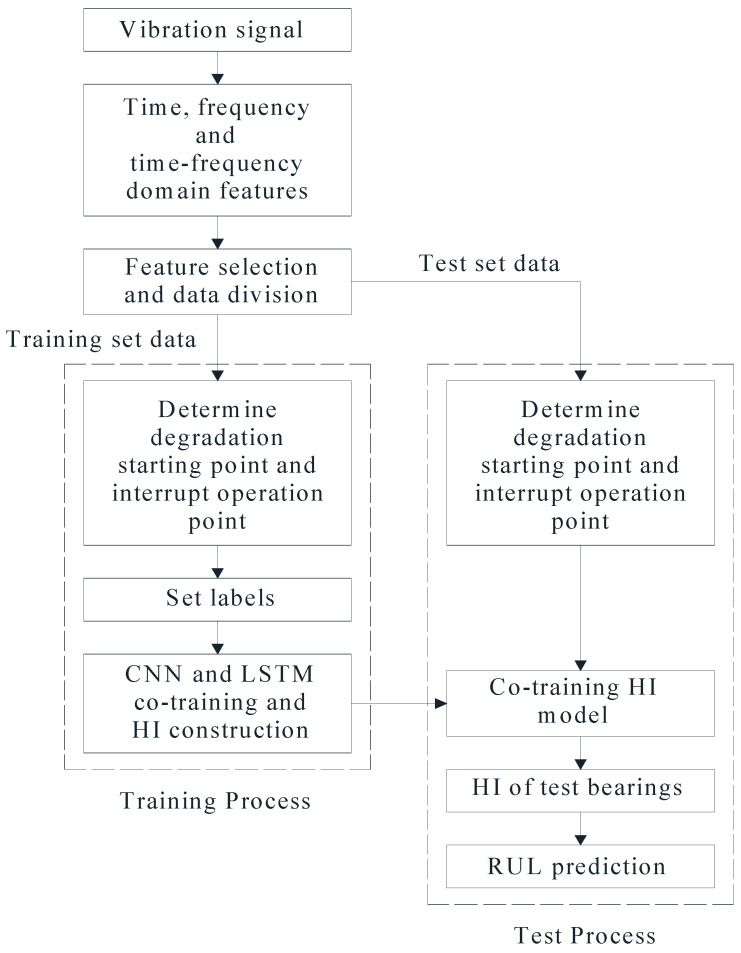
The process of cotraining-based RUL prediction of bearings.

The process of cotraining-based approach for RUL prediction of bearings is shown in Figure 6. The specific steps are as follows:

- Extracting time, frequency and time–frequency domain features from vibration signals, conducting feature selection to reduce the input data volume of the network, and splitting the data into training set and test set,
- Determining the degradation starting point and the interrupt operation point of the bearing according to the degradation stages.
- Cotraining CNN and LSTM with labeled training data to obtain the HI.
- Inputting the test set data into cotrained model to obtain the HI of test bearings and predicting their RUL.

## 4. Experimental Results

### 4.1. Experimental Results on Benchmark Dataset

#### 4.1.1. Dataset

Rolling bearings are an essential part of mechanical equipment. Although the bearing fault can be visually displayed in disassembly and replacement process, it is difficult to evaluate quantitatively the degree of failure and monitor the running status information in real time. Features extracted from vibration signal can determine the health status of rolling bearings.

**Figure 7:**
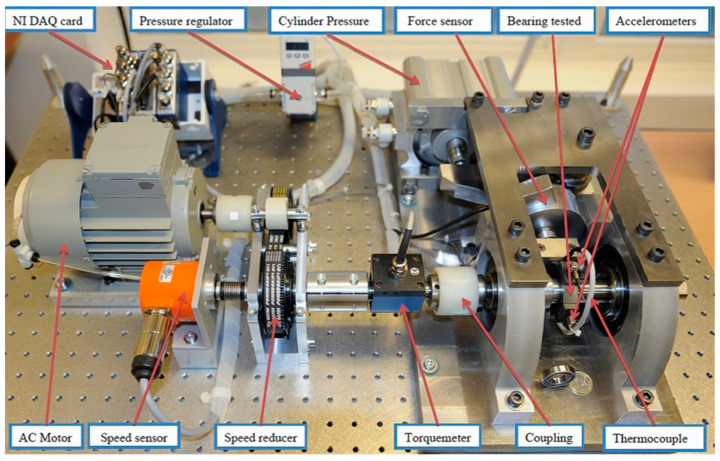
The experimental platform of PHM 2012.

The IEEE Reliability Institute and the FEMTO-ST Institute organized the IEEE PHM 2012 Data Challenge in 2012 [40]. The challenge provided a dataset for predicting the RUL of the bearings. In this paper, we used this dataset to train and evaluate the proposed approach. The experimental platform is shown in Figure 7.

**Figure 8:**
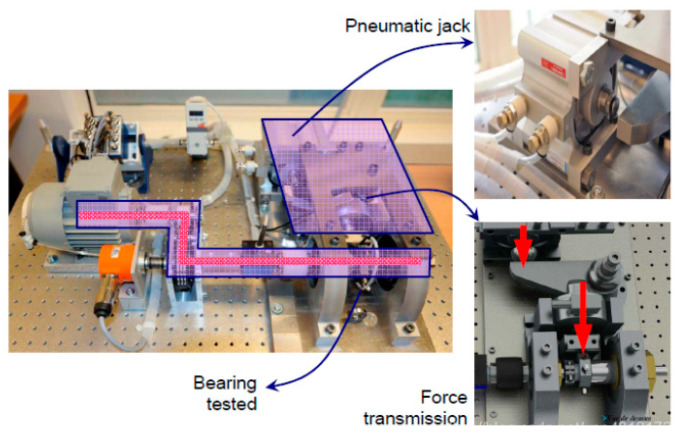
The load diagram of rotating part.

In the rotating part, the motor power is 250 W, and the maximum speed is 2830 rpm, which can ensure the speed of the second shaft is 2000 rpm. In the load part, this part is a pneumatic jack, which provides 4000 N dynamic load for the bearing. The load diagram is shown in Figure 8.

**Figure 9:**
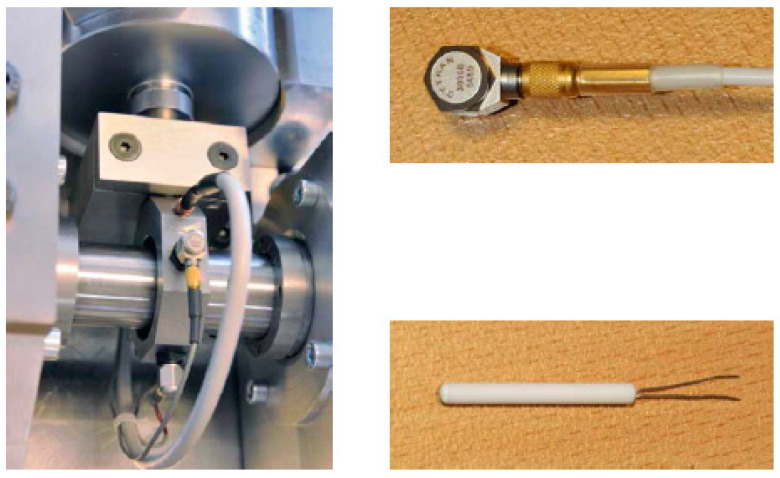
The vibration sensor.

In the testing part, the degradation data of bearing mainly consists of two parts, namely, vibration data and temperature data. The vibration sensor consists of two micro-accelerometers positioned at 90° to each other. The first one is placed on the vertical axis and the second one is placed on the horizontal axis. Two accelerometers are placed on the outer ring of the bearing along the radial direction, and the sampling frequency is 25.6 kHz. The temperature sensor is a resistance temperature detector placed in a hole close to the outer bearing ring with a sampling frequency of 0.1 Hz (Figure 9).

Therefore, the dataset includes three different working conditions:

- Load 4000 N, speed 1800 rpm
- Load 4200 N, speed 1650 rpm
- Load 5000 N, speed 1500 rpm

The specific training set and test set are shown in Table 1.

#### 4.1.2. Health Indicator (HI) Construction and Health Stage Division

Features extracted from vibration signals can determine the health status of rolling bearings. However, the original signal obtained from the sensor is the high-dimensional time-series data mixed with external noise, which makes it unsuitable for use directly in health-status monitoring. Feature extraction techniques and methods, in this case, can map the high-dimensional data into low-dimensional features to reduce the redundant information. Deep learning can extract deep features of degradation in vibration signal and avoid the interference of subjective factors from manual feature extraction.

In real-world tasks, there are numerous factors that affect the health status of the rolling bearing in different ways. If all the parameters are considered as the input of the network, problems such as training difficulties and overfitting may occur, resulting in inaccurate forecasting. In order to reduce the complexity of network training, the time, frequency and time–frequency domain-based features of the signal were selected first to reduce the input data volume of the network.

Time domain-based features include dimensional and dimensionless features. The dimensional feature can well represent the degradation trend of the rolling bearing but are not sensitive to rolling failure, which can be largely influenced by different working conditions. Dimensionless features are not significantly influenced by different working conditions and are more sensitive to rolling failure. If the signal sequence collected by sensor in each sampling time period is $x_{i}$, $x_{i}=\left[x_{1},x_{2},x_{3},\cdots ,x_{N}\right]$, where *N* is the number of sampling points, Table 2 and Table 3 give the dimensional and dimensionless features used in this paper.

Frequency domain-based features can reflect the failure type and the corresponding failure degree of the rolling bearing. These features of the signal are extracted from the spectral signal. Spectral signal is obtained by Fourier transformation of the time-domain signal, which describes the frequency components of original signal and the amplitude of each frequency component. Fast Fourier transformation (FFT) can reduce the amount of calculation and improve the calculation speed. In real-world tasks, FFT is often used to calculate the frequency spectrum of signal. The calculation formula is as follows:

(8) $$X_{N}\left(k\right)=\{\begin{array}{cc} X_{1}\left(k\right)+W_{N}^{k}X_{2}\left(k\right) & k=0,\cdots ,\frac{N}{2}-1\\ X_{1}\left(k-\frac{N}{2}\right)-W_{N}^{k}X_{2}\left(k-\frac{N}{2}\right) & k=\frac{N}{2},\cdots ,N-1 \end{array}$$

where $X_{1}\left(k\right)$ is the discrete Fourier transform of the even items in the time-series x(i), and $X_{2}\left(k\right)$ is the discrete Fourier transform of the odd items.

If the sampling frequency of the original signal is $F_{S}$, after FFT, the frequency of the *k*th frequency point in the spectrum is $f_{k}=\left(k-1\right)*F_{s}/N$. Since the signal spectrum is obtained, frequency domain-based features can be obtained from the spectrum. Table 4 gives the frequency domain-based features used in this paper.

**Figure 10:**
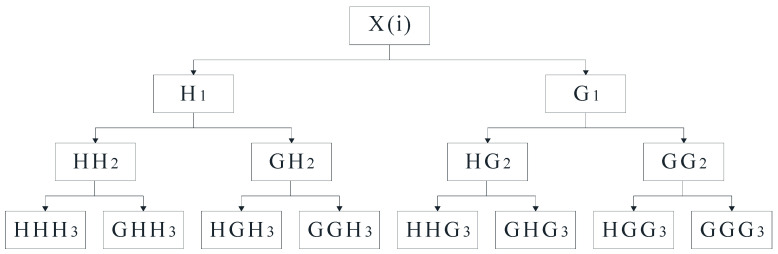
The structure of three-layer wavelet packet decomposition.

Time domain- and frequency domain-based features can intuitively show parts of the inherent information of the original signal. However, since original vibration signal of the rolling bearing is a nonstationary signal, it is difficult to accurately describe the change law of original signal only using time domain- and frequency domain-based features. The time–frequency analysis method is introduced in this case to analyze the original signal. The wavelet packet decomposition is a commonly used time–frequency analysis method, which can well analyze nonlinear nonstationary signals. Compared with wavelet decomposition, wavelet packet decomposition can decompose both the low-frequency and the high-frequency part of the signal. Original signal after wavelet packet decomposition will be decomposed into each sub-band, so as to realize further time–frequency localized analysis. Figure 10 shows the structure of three-layer wavelet packet decomposition, where x(i) denotes the original vibration signal, *H* denotes the low-frequency component, and *G* denotes the high-frequency component.

The original signal is decomposed into two parts, high frequency and low frequency, through wavelet packet decomposition. The feature information of the original signal is retained while obtaining the deep information, which is beneficial to the analysis of nonstationary signals. The base wavelet has great influence on feature extraction of the bearing. In this paper, we select the base wavelet according to the variation rate of energy fluctuation. First, the energy of each band is calculated as a percentage of the overall signal energy $E_{j}^{n}$, where $n=1,\;2,\;\cdots ,\;2^{j}$, and then the energy fluctuation parameter of $E_{flu}$ is defined as:

(9) $$E_{flu}=\frac{max\;\left(E_{j}^{n}\right)-mean\;\left(E_{j}^{n}\right)}{max\;\left(E_{j}^{n}\right)-min\;\left(E_{j}^{n}\right)}\;$$

As can be seen from the equation, calculate the $E_{flu}$ is a normalized gauge, and the value of $E_{flu}$ is between [0, 1] as the signal changes during transmission. The energy distribution is more uniform in the actual health state of the bearings, while the energy distribution is unbalanced in the fault state, and the two values may differ significantly. Therefore, in order to make the data in different states comparable, based on Equation (9), we can calculate the energy function parameters corresponding to the vibration signal under normal and fault state of the bearing: $E_{nor}$, $E_{fau}$, then calculate the rate of change of energy fluctuations:

(10) $$E^{\prime }=\frac{\left(E_{fau}-E_{nor}\right)}{E_{nor}}\times 100\%$$

The larger $E^{\prime }$ is, the more the features of the fault signal deviate from the normal signal and the better the fault feature extraction is. Wavelet basis function corresponding to $E_{max}^{\prime }$ is the most optimal wavelet basis function for decomposing the bearing signal using wavelet packets.

In this paper, we choose db3, db8, haar and db4 for comparison. The respective energy fluctuation parameters and the rates of change are shown in Table 5. It can be seen from the table that the maximum energy fluctuation rate of change $E_{max}^{\prime }$ is the haar wavelet basis function, so it is the optimal wavelet basis function for the decomposition of the bearing signal.

We use haar wavelet basis function to decompose the signal of the bearing with three layers of wavelet packets. Eight sub-bands are obtained and the energy ratio of the sub-bands is used as the time–frequency based feature. The energy feature of each sub-band is defined as follows:

(11) $$E_{j}^{l}=\overset{n}{\underset{i=1}{\sum }}\left(x_{j}^{l}{\left(i\right)}^{2}\right)$$

where *j* denotes the number of decomposition layers, *l* denotes the number of nodes obtained by decomposing each layer, and *n* denotes the length of the node signal $x_{j}^{l}\left(i\right)$. The energy ratio after wavelet packet decomposition is:

(12) $$P_{j}^{l}=\frac{E_{j}^{l}}{\sum_{l=0}^{2^{j}-1}E_{j}^{l}}$$

**Figure 11:**
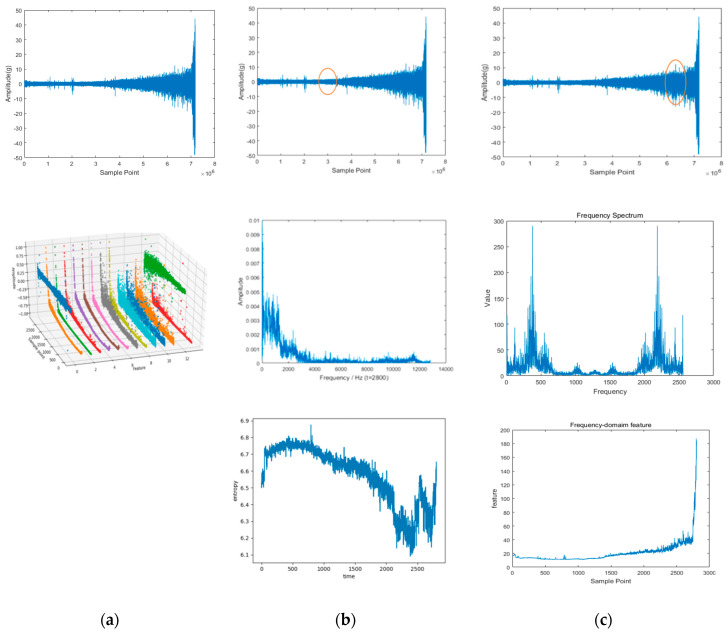
Time domain, frequency domain, and time–frequency domain−based features of the signal (bearing 1-1): (**a**) time domain-based features; (**b**) time–frequency domain based features; (**c**) frequency domain-based features.

According to the relevant research, when using vibration signals to track bearing degradation, the horizontal vibration signals often contain more degradation information than the vertical vibration signals. In this paper, we use only the horizontal vibration signals of bearings as experimental data for subsequent research. Taking the bearing 1-1 as an example, Figure 11 shows a schematic diagram of the time-domain, frequency-domain, and time–frequency domain features of the horizontal vibration signal. After all features are extracted, the features need to be standardized by the following formula:

(13) $$x_{i}=\frac{2x_{i}-max\left(x\right)-min\left(x\right)}{max\left(x\right)-min\left(x\right)}$$

However, not all of the above feature parameters can better reflect the degradation state of the bearing. In order to prevent redundant features from having a negative impact on the accurate evaluation of the bearing degradation state, the above feature parameters need to be further screened to remove the feature parameters that are not sensitive to the bearing state. In general, the feature parameters that can better describe the bearing degradation state should have good monotonicity, robustness and high correlation with the bearing degradation process. At the same time, they should have a certain ability to identify different stages of bearing degradation. Therefore, we choose monotonicity, correlation, robustness and identifiability as indicators to further screen the selected feature parameters. At the same time, in order to more comprehensively evaluate the sensitivity of the degradation features, the four evaluation indicators are linearly combined to obtain a comprehensive indicator, and the formula is as follows:

(14) $$F\left(X\right)=w_{1}Mon\left(X\right)+w_{2}Corr\left(X,T\right)+w_{3}Rob\left(X\right)+w_{4}Ide\left(X,C\right)$$

where *T* is the time, *C* is the life stage of the bearing ($C=\left[C_{1},\;C_{2},\;\cdots C_{n}\right]$), and $w_{1}$, $w_{2}$, $w_{3}$, $w_{4}$ is the corresponding weights of monotonicity index, correlation index, robustness index and identifiability index, respectively. The larger the value of comprehensive index *F*, the more sensitive the selected feature is to the degradation process of bearings.

**Figure 12:**
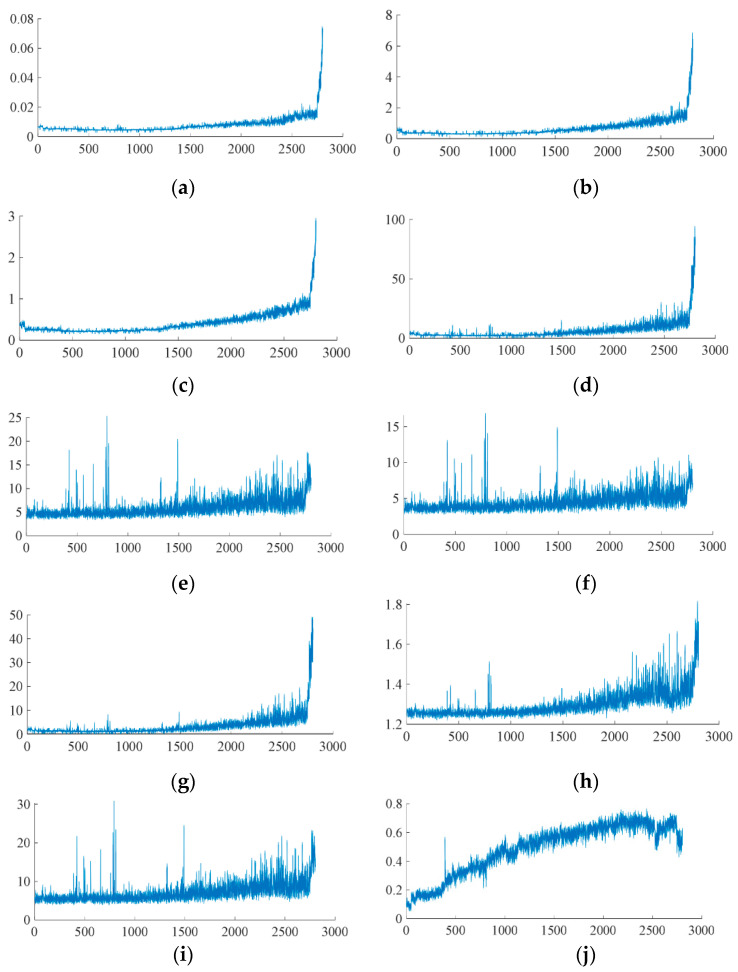
The obtained features of bearing 1-1 (10 s): (**a**) frequency-domain amplitude average, (**b**) root mean square, (**c**) square root amplitude, (**d**) peak-to-peak value, (**e**) impulse factor, (**f**) peak value factor, (**g**) kurtosis factor, (**h**) peak value, (**i**) waveform factor, (**j**) first frequency sub-band energy ratio of the three-layer wavelet packet decomposition.

Since the degradation process of bearings is a monotonous and irreversible process, the selected degradation features need to reflect the overall degradation trend of bearings, so the monotonicity of degradation features should occupy a relatively large weight in the comprehensive index. At the same time, in the subsequent experiments, we found that most of the robustness indicators of the extracted features are at a relatively high level, which reduces the discrimination of the robustness indicators on such features and is not conducive to the screening of degradation features. Therefore, the weight of the robustness indicators in the comprehensive indicators should be reduced. To sum up, we set the weights of the monotonicity, correlation, robustness and identifiability indicators in the comprehensive indicators to 0.4, 0.2, 0.2 and 0.2 respectively. We screen the degradation features under each working condition and select the first 10 features with the largest comprehensive index to build the degradation feature set. The degradation features we finally obtained include: (1) frequency-domain amplitude average, (2) root mean square, (3) square root amplitude, (4) peak-to-peak value, (5) impulse factor, (6) peak value factor, (7) kurtosis factor, (8) peak value, (9) waveform factor, (10) first frequency sub-band energy ratio of the three-layer wavelet packet decomposition. Taking the bearing 1-1 as the example, the obtained features are shown in Figure 12.

Theoretically, the nondegraded stage of the rolling bearing can hardly provide any degradation information, and the monitoring signal of the nondegraded stage and the degraded stage are two independent distributions. The training set of the neural network is not suitable for two or more distributions, so the neural network is not adoptable to learn the information from the nondegraded stage. According to the degradation trend of the bearing during the whole operating time, this paper divides bearing degradation process into three stages: stable degradation, rapid degradation, and rapid failure period.

- Stable degradation period: the rolling bearing is in normal operating conditions, but the degradation has begun and will continue, and the signal features of degradation are not obvious;
- Rapid degradation period: the operation of the rolling bearing becomes more and more unstable, and the signal features of degradation are extremely obvious;
- Rapid failure period: it is the period from rapid degradation to bearing failure, the features of failure are extremely obvious.

Three stages of bearing degradation process are defined as follows:

(15) $$T=\{\begin{array}{cc} \mathrm{Stable}\;\mathrm{degradtion}\;\mathrm{period} & 0<t<t_{0}\\ \mathrm{Rapid}\;\mathrm{degradtion}\;\mathrm{period} & t_{0}<t<t_{1}\\ \mathrm{Rapid}\;\mathrm{failure}\;\mathrm{period} & t_{1}<t \end{array}$$

**Figure 13:**
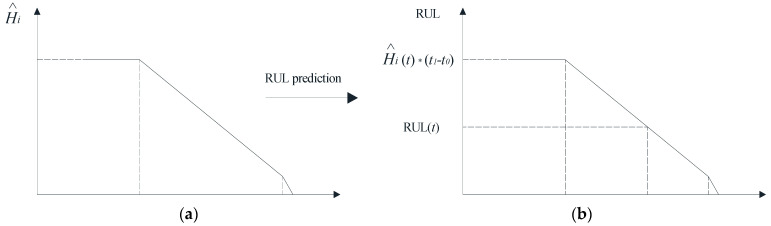
RUL prediction of rolling bearing: (**a**) the HI of the test bearing; (**b**) RUL prediction.

In the testing phase of the model, the HI of the test rolling bearing can be obtained by inputting test set rolling bearing features into the trained network model, as shown in Figure 13a. In the figure, $\hat{t_{0}}$ denotes the predicted degradation starting value, and ${\hat{H}}_{i\;}\left(t\right)$ denotes the rolling bearing HI constructed by the predictive model. From the obtained rolling bearing HI, RUL prediction can be achieved (Figure 13b). Generally, the rolling bearing RUL prediction can be divided into three stages. The first period is before the degradation starting point, the rolling bearing is in a healthy status in this period, only general attention is required, and the RUL index remains relatively stable (we generally define this period as when the RUL ≥ 55%). The second period is after the bearing enters the rapid degradation period, the RUL decreases with the reduction of HI, and it is a period that requires careful attention. The third period is when the rolling bearing enters the rapid failure period (we generally define this period as when the RUL ≤ 5%). Some unstable changes may occur at any time, and it is prone to catastrophic damage if the rolling bearing continues to operate.

The bearing degradation starting point needs to be determined first to construct a more accurate HI model. The degradation starting point of the rolling bearings in PHM 2012 dataset under three operating conditions is shown in Table 6.

**Figure 14:**
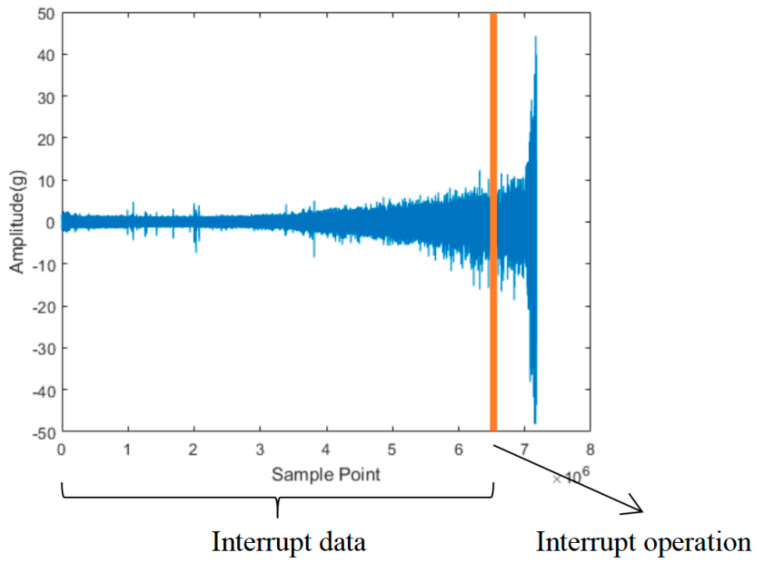
Interrupt data (unlabeled).

It can be seen from the table that the degradation starting point of the bearing can be varied due to the influence of various operating conditions. The earliest and latest degradation starting points are at 7% and 94% of the whole bearing life cycle. In order to improve RUL prediction efficiency, we set the interrupt operation of the bearing at 95% of the whole life cycle (Figure 14). The bearing that exceeds 95% of its whole life cycle is seen as entering the rapid failure period. Taking the degradation signal features from 5% to 95% of its whole life cycle as the dataset and the degradation percentage as RUL output label.

#### 4.1.3. Comparison and Analysis of RUL Prediction Results

In this paper, a CNN and an LSTM are trained separately using a small quantity of labeled data. The two networks are then cotrained on large quantities of unlabeled data, adding unlabeled samples with high confidence to each other’s training sets to obtain rolling bearing HI. Finally, the monitoring data are input into HI model to obtain the HI of the monitoring bearing, and the RUL prediction of the monitoring rolling bearing is realized. The network parameters of CNN and LSTM are shown in Table 7 and Table 8.

Various indicators can evaluate the performance of RUL prediction. In this paper, the root mean square error (*RMSE*) and the mean absolute percentage error (*MAPE*) are selected to evaluate the prediction results, which are calculated as follows:

(16) $$RMSE=\sqrt{\frac{1}{n}\sum_{n-1}^{n}{\left(y_{i}-y_{pre}\right)}^{2}}$$

(17) $$MAPE=\frac{100}{n}\sum_{i=1}^{n}\left|\frac{y_{i}-y_{pre}}{y_{i}}\right|$$

where $y_{i}$ and $y_{pre}$ denote the prediction result and the true value at moment *i*, and *n* denotes the number of samples in the test set.

We first compare the prediction results of proposed approach with individual CNN and LSTM under same operation condition. Taking the rolling bearing under operation condition 1 as an example, bearing 1-1 and bearing 1-2 are selected as the failure data (with labels), and the remaining bearings as the interrupt data (unlabeled). Interrupt the data at 95% of the whole life cycle, and add one rolling bearing at a time as interrupt training data. CNN and LSTM are trained separately using the small quantity of labeled data. The two networks are then cotrained on large amounts of unlabeled data, adding unlabeled samples with high confidence to each other’s training sets to obtain rolling bearing HI. Finally, the monitoring data are input into HI model to obtain the HI of the monitoring bearing, and the RUL prediction of the monitoring rolling bearing is realized.

**Figure 15:**
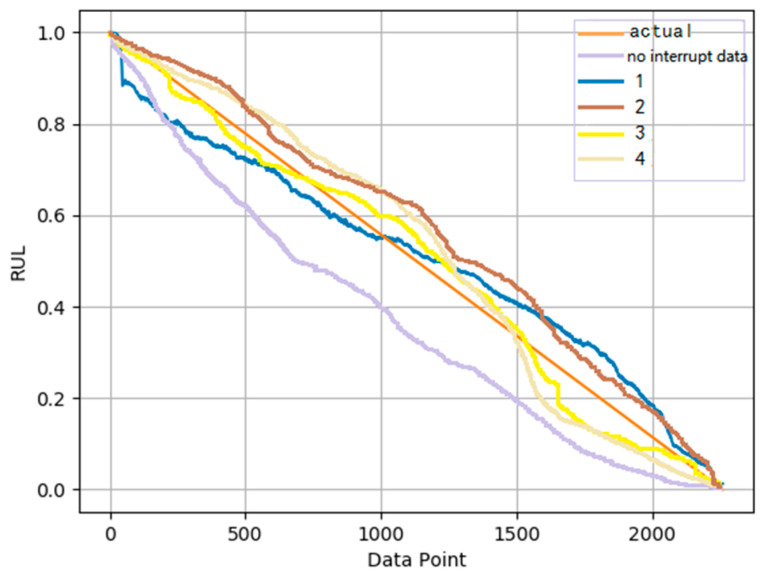
RUL prediction vs. true RUL (under operation condition 1).

The RUL prediction curves obtained by continuously increasing interrupt training data are compared with the actual RUL in Figure 15. It can be seen from the figure that by increasing interrupt training data, the RUL prediction curve is much closer to the true RUL. It demonstrated that the learning performance of cotraining can be improved by continuously increasing the training data. It also verifies that when using semisupervised learning for prediction tasks, within a certain range, the higher percentage of the data is used for training, the better the results will obtain.

The RMSE and MAPE values of the test set on CNN, LSTM, and the proposed cotraining are provided in Table 9. It can be seen from the table that CNN performs better than LSTM in RUL prediction with a small quantity of labeled data, and the cotraining can obtain better prediction results than individual CNN and LSTM. Meanwhile, by adding interrupt training data, the RUL prediction results can be further improved. However, it is worth noting that the RMSE and MAPE value of cotraining become larger after adding four interrupt training data compared to adding three interrupt training data. A reasonable explanation is that the accuracy of RUL prediction has entered a “bottleneck.” Generally, the effective method to improve prediction accuracy is mainly to increase or replace variables rather than expanding the training sample size.

For comparison and analysis, the RUL prediction result under different operation conditions, bearing 1-3, 2-3 and 3-3 are selected as the test bearings, and the remaining 14 bearings are the training bearings. The RMSE and MAPE of training process and testing process are shown in Table 10 and Table 11, namely, the test bearing HI construction error and the test bearing RUL prediction error.

Since there are few publications are available about cotraining-based approaches for RUL prediction. In order to verify the improvements of the proposed approach, this paper compares the RUL prediction results of the proposed cotraining with the SAE+LSTM stacking network [41] and the CNN+LSTM stacking network [38] in the existing literature using RMSE on PHM 2012 dataset. By taking the bearing 2-2 as the example, the RMSE value of the SAE+LSTM stacking network is 137.12, the RMSE value of the CNN+LSTM stacking network is 49.36, and the RMSE value of the proposed cotraining is 54.72. It is obviously that the RUL prediction result of the proposed method is significantly better than that of the SAE+LSTM stacking network. Compared with the CNN+LSTM stacking network, the RMSE value is slightly higher. The reason is that there are enough training samples in the dataset, and the deep degradation features obtained from supervised learning can better reflect degradation process of the rolling bearing. However, the proposed method can obtain similar RUL prediction results with supervised learning by artificially setting a small number of labeled training samples. Therefore, the proposed approach in this paper is effective.

### 4.2. Experimental Results on Real-World Cases

#### 4.2.1. Case Description

**Figure 16:**
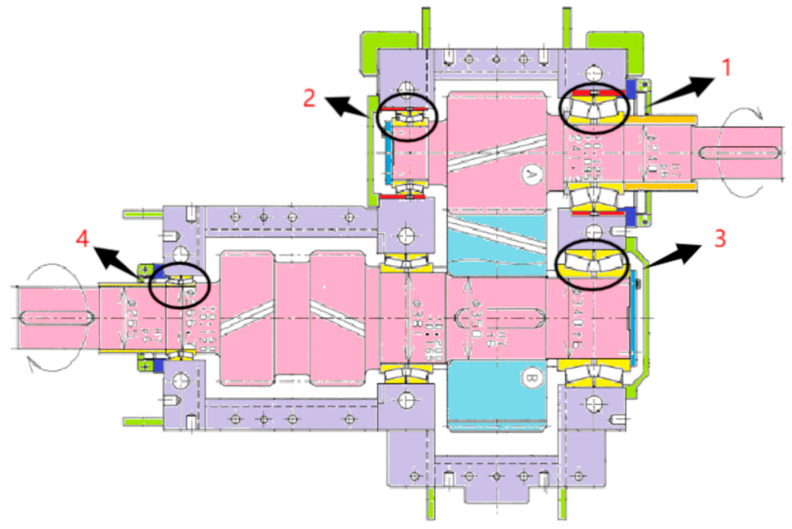
The structure of the gearbox (1, 2, 3, 4 represents 4 bearings respectively).

**Figure 17:**
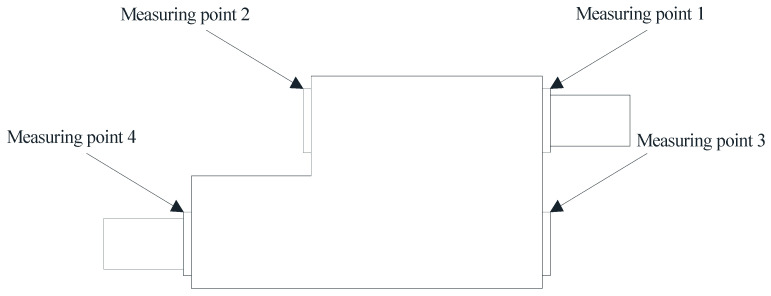
The layout of the vibration measuring points.

The pickling line five-stand unit is the crucial production equipment in the Second Silicon Steel Plant of Wuhan Iron and Steel (Group) Company, China. Due to congenital equipment defects, the failure of burning bearings often occur, and the unit was forced to shut down for unplanned maintenance, which may seriously affect safe production. In response to this urgent problem, the regular on-site maintenance was conducted for precision testing of the gearbox, collecting vibration data and applying the proposed approach to predict the RUL of bearings. As a consequence, the bearings were replaced before they entered the rapid failure period, and the predictive maintenance was achieved, which effectively avoided the occurrence of accidents. The structure of the gearbox is shown in Figure 16 and the layout of the vibration measuring points is shown in Figure 17. In the past, burning bearing accidents had occurred at the position of measuring point 1, which was the focus of attention.

#### 4.2.2. Vibration Signal Test Process and RUL Prediction

**Figure 18:**
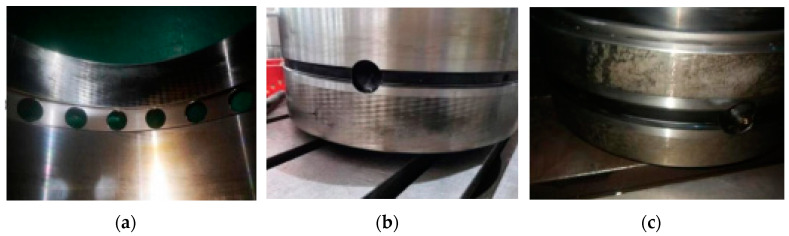
The actual status of the bearing offline: (**a**) bearing eccentric sleeve wear; (**b**) bearing outer ring impact marks; (**c**) bearing inner ring wear.

The vibration signal testing process and RUL prediction results are presented in Table 12. According the research object, we set the bearing that exceeds 97% of its whole life cycle as entering the rapid failure period, take the degradation features from 50% to 97% of its whole life cycle as the dataset and degradation percentage as the RUL output label. In order to verify the effectiveness of the proposed approach, the disassembled offline bearings’ health-state judgment was adopted for comparison. It can be seen from the offline bearing that the eccentric sleeve was severely worn, the inner ring gap was large, the outer ring had impact marks, and the inner ring was severely worn. The bearing could be burnt or broken at any time, which was in good agreement with RUL prediction results. The example diagrams of the offline bearing are shown in Figure 18.

## 5. Conclusions

This paper innovatively proposed a cotraining based semisupervised approach for RUL prediction of bearings. In this approach, a CNN and an LSTM are first cotrained on large quantities of unlabeled data by adding unlabeled samples with high confidence to each other’s training set to obtain the health indicator (HI), then the monitoring data are input into HI and RUL prediction is realized. The RMSE and MAPE value are used and the IEEE PHM 2012 dataset is adopted to realize the improvement of the proposed approach. The results show that the proposed approach have confirmed the validity of the cotraining-based approach in RUL prediction of bearings. However, due to the limitations of network selection and parameter settings, the approach still has some room for improvement. Further research can be carried on the following:

- In this paper, a CNN and an LSTM were selected as the initial network for cotraining. Further work can be carried out based on combing different networks for cotraining, comparing the RUL prediction results of different combinations, and exploring the superiority and general rules of different combinations in various application scenarios.
- In this paper, only two networks were used for cotraining. Further work can be carried out based on increasing the number of cotraining networks to cotraining multiple networks, and integrating multiple prediction results.

## Figures and Tables

**Table 1:** The training set and test set in PHM 2012.

Datasets	Operation Conditions		
	Condition 1	Condition 2	Condition 3
Training set	Bearing 1-1	Bearing 2-1	Bearing 3-1
	Bearing 1-2	Bearing 2-2	Bearing 3-2
Test set	Bearing 1-3	Bearing 2-3	Bearing 3-3
	Bearing 1-4	Bearing 2-4	
	Bearing 1-5	Bearing 2-5	
	Bearing 1-6	Bearing 2-6	
	Bearing 1-7	Bearing 2-7	

**Table 2:** Dimensional time domain-based features.

No.	Feature	Expression
1	Root Mean Square Value	$X_{RMS}=\sqrt{\frac{1}{N}\overset{N}{\underset{i=1}{\sum }}x_{i}^{2}}$
2	Mean Value	$\bar{X}=\frac{1}{N}\overset{N}{\underset{i=1}{\sum }}x_{i}$
3	Standard Deviation	$X_{\sigma }=\sqrt{\frac{1}{N}\overset{N}{\underset{i=1}{\sum }}{\left(x_{i}-\bar{X}\right)}^{2}}$
4	Square Root Amplitude	$X_{r}={\left[\frac{1}{N}\overset{N}{\underset{i=1}{\sum }}\sqrt{\left|x_{i}\right|}\right]}^{2}$
5	Absolute Mean Amplitude	${\bar{X}}_{p}=\frac{1}{N}\overset{N}{\underset{i=1}{\sum }}\left|x_{i}\right|$
6	Peak Value (Maximum Value)	$X_{max}=max\left\{\left|x_{i}\right|\right\}$
7	Peak-to-Peak Value	$X_{p\text{-}p}=max\left(x_{i}\right)-min\left(x_{i}\right)$

**Table 3:** Dimensionless time domain-based features.

No.	Feature	Expression
1	Skewness	$X_{ske}=\frac{\sum_{i=1}^{N}{\left(x_{1}-\bar{X}\right)}^{3}}{\left(N-1\right)X_{\sigma }^{3}}$
2	Kurtosis	$X_{kur}=\frac{\sum_{i=1}^{N}{\left(x_{1}-\bar{X}\right)}^{4}}{\left(N-1\right)X_{\sigma }^{4}}$
3	Skewness Factor	$I_{ske}=\frac{X_{ske}}{X_{RMS}^{3}}$
4	Kurtosis Factor	$I_{kur}=\frac{X_{kur}}{X_{RMS}^{4}}$
5	Peak Value Factor	$I_{p}=\frac{X_{max}}{X_{rms}}$
6	Impulse Factor	$I_{i}=\frac{X_{max}}{{\bar{X}}_{p}}$
7	Waveform Factor	$I_{w}=\frac{X_{rms}}{{\bar{X}}_{p}}$
8	Margin Factor	$I_{m}=\frac{X_{max}}{X_{r}}$

**Table 4:** Frequency domain-based features.

No.	Feature	Expression
1	Gravity Frequency	$S_{FC}=\frac{\sum_{k=0}^{N-1}f_{k}X\left(k\right)}{\sum_{k=0}^{N-1}X\left(k\right)}$
2	Frequency-Domain Amplitude Average	$\bar{S}=\frac{1}{N}\overset{N-1}{\underset{k=0}{\sum }}X\left(k\right)$
3	Frequency-Domain Standard Deviation	$S_{RVF}=\sqrt{\frac{\sum_{k=0}^{N-1}{\left(f_{k}-\bar{S}\right)}^{2}X\left(k\right)}{\sum_{k=0}^{N-1}X\left(k\right)}}$
4	Root-Mean-Square Frequency	$S_{RMSF}=\sqrt{\frac{\sum_{k=0}^{N-1}f_{k}^{2}X\left(k\right)}{\sum_{k=0}^{N-1}X\left(k\right)}}$

**Table 5:** Energy fluctuation parameters and the rates of change.

Wavelet Function	Energy Fluctuation Parameters		Rate of Change $E^{\prime }$/%
db3	Normal	0.48	77.47
	Fault	0.86	
db8	Normal	0.51	67.48
	Fault	0.86	
haar	Normal	0.47	87.41
	Fault	0.86	
db4	Normal	0.49	75.70

**Table 6:** The degradation starting point of the rolling bearing in PHM 2012 dataset.

Bearing	Degradation Starting Point (10 s)	Bearing	Degradation Starting Point (10 s)	Bearing	Degradation Starting Point (10 s)
1-1	1325	2-1	875	3-1	491
1-2	827	2-2	195	3-2	1585
1-3	1352	2-3	1946	3-3	312
1-4	1083	2-4	742		
1-5	2410	2-5	2263		
1-6	2415	2-6	686		
1-7	2198	2-7	222		

**Table 7:** The training parameters of CNN.

Network Layers	Size of Convolution Kernels	Number of Convolution Kernels	Output Size
Conv1	101×1	8	1180×8
Pool1	10×1	/	118×8
Conv2	51×1	16	68×16
Pool2	10×1	/	7×16
FL1	40	/	40×1
FL2	10	/	10×1
FL3	1	/	1

**Table 8:** The training parameters of LSTM.

Parameters	Size of Convolution Kernels
Layers	4
Learning rate	0.0006
Hidden unit	200
Time step	30
Batch size	50

**Table 9:** Comparison of RMSE and MAPE values in CNN, LSTM and Cotraining CNN+LSTM.

Interrupt Training Data	Evaluation Indicator	CNN	LSTM	Cotraining
None	RMSE	83.44	79.48	78.05
	MAPE	19.80	17.65	17.01
1	RMSE	-	-	63.41
	MAPE	-	-	13.56
2	RMSE	-	-	58.19
	MAPE	-	-	14.33
3	RMSE	-	-	55.06
	MAPE	-	-	13.68
4	RMSE	-	-	55.73
	MAPE	-	-	13.89

**Table 10:** Test bearing HI construction error.

Bearings	RMSE	MAPE
Bearing 1-3	0.0765	0.0489
Bearing 2-2	0.0724	0.0563
Bearing 3-3	0.0420	0.0267

**Table 11:** Test bearing RUL prediction error.

Bearings	RMSE (10 s)	MAPE (10 s)
Bearing 1-3	59.33	19.31
Bearing 2-2	54.72	11.06
Bearing 3-3	4.11	5.70

**Table 12:** The vibration test process and RUL prediction results.

Test Time	Time−Domain Waveform of Measuring Point 1	Spectrogram of Measuring Point 1	RUL Prediction
1 June 2021	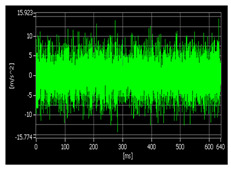	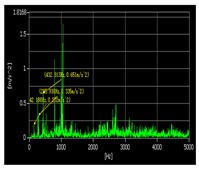	The bearing is in the stable degradation period
18 February 2022	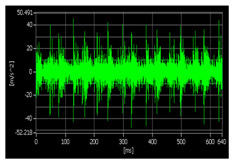	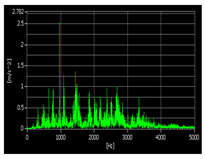	The bearing is in the rapid degradation period (85% of the whole life cycle, RUL = 12%)
23 May 2022	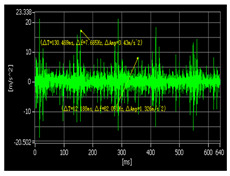	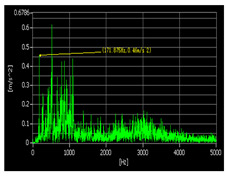	The bearing is in the rapid failure period. (RUL < 5%)
